# Field evaluation of a novel synthetic odour blend and of the synergistic role of carbon dioxide for sampling host-seeking *Aedes albopictus* adults in Rome, Italy

**DOI:** 10.1186/s13071-014-0580-9

**Published:** 2014-12-11

**Authors:** Marco Pombi, Frans Jacobs, Niels O Verhulst, Beniamino Caputo, Alessandra della Torre, Willem Takken

**Affiliations:** Dipartimento di Sanità Pubblica e Malattie Infettive, Università di Roma “Sapienza”, Rome, Italy; Laboratory of Entomology, Wageningen University and Research Centre, Wageningen, The Netherlands

**Keywords:** *Aedes albopictus*, Mosquito, Adult collections, BG-sentinel trap, Attraction, Odour bait, CO_2_, Synergism

## Abstract

**Background:**

Despite the expanding worldwide distribution of *Aedes albopictu*s and its increasing relevance as arboviral vector, current methods to collect adult specimens are not optimal. Improved approaches are thus needed to monitor their density and pathogen infections, and to establish baseline data for control interventions. A widely used device is the BG-Sentinel (BG-trap) which mostly targets host-seeking females attracted by release of CO_2_ and/or a synthetic odour blend (the BG lure). We compared the attractiveness of this blend to that of the Mbita (MB5) lure, a new synthetic blend of proven efficiency in attracting Afrotropical malaria vectors, and evaluated the additional effect of CO_2_ to the two odour baits.

**Findings:**

We carried out 6x6 Latin square experiments in two *Ae. albopictus*-infested areas in Rome, baiting the BG-traps as follows: CO_2_, BG lure, MB5 lure, BG lure + CO_2_, MB5 lure + CO_2,_ no bait. CO_2_ was derived from yeast-fermented sugar. Overall, 949 females and 816 males were collected. Baited traps collected significantly more females than unbaited ones. Traps baited with either lures in combination with CO_2_ were more effective than those baited with CO_2_ alone. No significant differences were observed in female captures between traps baited with any of the two lures, nor between the two lures, independently from the addition of CO_2_. The use of BG lure + CO_2_ significantly increased males catches compared to unbaited traps.

**Conclusions:**

The results suggest a broad significance of the MB5 lure for sampling medically important mosquito species and highlight the high efficacy of the combination of lures + CO_2_ for female *Ae. albopictus* and of BG lure + CO_2_ for males, leading to consider CO_2_ as an essential additional cue for the sampling of this species.

## Findings

*Aedes albopictus* is an aggressive daytime-biting mosquito responsible for prominent Chikungunya virus epidemics in the Indian Ocean [1]. Moreover during the past 20 years, the species became a major nuisance in Italy as well as in other southern European countries, where it has also been responsible for endemic Chikungunya transmission in Italy as well as dengue in mainland France and Croatia [2].

While ovitraps are considered the best approach to detect the presence of new establishments of *Ae. albopictus*, current methods to collect adult specimens in order to better monitor their density and their possible infections with mosquito-borne pathogens, and to establish baseline data for control interventions and assess their efficacy, are not optimal [3]. Presently, the most widely used trap to collect host-seeking *Ae. albopictus* females is the BG-Sentinel trap (hereafter BG-trap; Biogents A.G., Regensburg, Germany). This device consists of a cylindrical counterflow trap generally baited with a slow-release pack of a synthetic attractant (BG lure). The fan blows downward into the cylinder so that the attractant is blown upward, by overpressure, and out through the upper netting cover. Mosquitoes attracted to the source of odour are drawn into a mesh collecting bag by the downward airflow originating from the fan. Although the BG-trap was originally developed to collect the main dengue vector species *Ae. aegypti* in tropical areas [4-6], field studies have shown its efficacy in collecting other mosquito species as well, particularly when simultaneously releasing both BG lure and CO_2_, in North America [7] as well as in Europe [8,9].

A recent study showed a very high attractiveness of selected synthetic blends simulating human odours to host-seeking Afrotropical malaria vectors as well as the filariasis vector *Culex quinquefasciatus* and other potential mosquito species [10]. Here we report results of field experiments carried out in Rome, Italy, to test the efficacy of one of these blends as a possible alternative to the commonly used BG lure to collect adult *Ae. albopictus* and to assess the possible effect of CO_2_ addition to the lures.

## Material and methods

Two 6 × 6 Latin square experiments were carried out from 5 to 13 October 2011 in two highly *Ae. albopictus*-infested areas in Rome (~3000 m^2^ each and ~1 km distant one from the other), i.e. the garden of the Unit of Comparative Anatomy of the Department of Biology and Biotechnology of Sapienza University [11] and Verano Cemetery [12]. At each location, six BG-Sentinel traps (Biogents GmbH, Regensburg, Germany) were located at approximately 20 m from each other and baited with five different odour blends, as follows: 1- CO_2_ (produced by 17.5 g of fermenting baker’s yeast in a sucrose solution 0.29 M, as described by Smallegange et al. [13]). 2- BG lure, i.e. standard BG lure sachets, Biogents, consisting of NH_3_, L-Lactic acid and hexanoic acid in unknown concentrations. 3- Mbita blend, i.e. MB5 lure, NH_3_ (25% in water), L-(+)-Lactic acid (88-92% in water), tetradecanoic acid (16% in EtOH), 3-methyl-1-butanol (0.01% in paraffin oil) and butan-1-amine (0.001% in paraffin oil) [14]. One mL of each of the solutions was separately embedded in nylon strips (26.5 × 1.0 cm) mounted on a wire frame and placed at the entrance of BG trap [15]. 4- BG lure + CO_2_. 5- MB5 lure + CO_2_. 6- a trap activated without any odour and used as a negative control (no bait). Temperature and relative humidity data of the sampling period in the Municipality of Rome were downloaded by the website Archivio Meteo Italia (http://archivio-meteo.distile.it); no rain occurred during the period of the experiment. Every 24 h, all BG-traps were rotated clockwise to the next position for a total of six days. Mosquitoes during each 24 h-sampling were removed from the trap-bags and frozen. Morphological identifications were carried out following Severini et al. [16].

For both *Ae. albopictus* males and females collected, data were analysed by Generalized Linear Model (GLM) with Poisson distribution with log-link function, dispersion parameter estimated using sampling area, days of collection, trap positions and bait as factors. The final model, including significant factors, was used to calculate the estimated mean trap catches and standard errors followed by a pairwise comparison with LSD correction. All analyses have been performed with SPSS statistical software [17].

## Results and discussion

A total of 1,858 mosquitoes were collected (1,191 in the Anatomy garden and 667 in the Verano cemetery): 95% of these were *Ae. albopictus* and 5% *Culex pipiens*. Table 1 shows the median and total catches of *Ae. albopictus* females and males for each site and for each tested odour blend. Overall, a comparable number of females were collected in the two areas, while a significantly higher number of males were collected in the Anatomy garden (i.e. males:females ratio was 1.5 in the Anatomy garden and 0.3 in Verano; *χ*^2^ = 229.1; p < 0.0001). Since there is no reason to assume that this reflects a real difference in sex-ratio in the two areas, the observed difference may be due to their distinctive ecological features. In particular, it may be hypothesized that the lower abundance of visual landmarks for male swarming [18] in Anatomy garden (as opposed to Verano cemetery where landmarks such as graves or trees are very abundant) makes the white/dark contrast of BG-Sentinel trap the major swarming landmark in this area [19,20].

**Table 1:** **Descriptive statistics of** ***Aedes albopictus*** **collected with different odour blends**

**Bait**	**Sex**	**Anatomy garden**			**Verano cemetery**		
		**Median**	**5-95% Percentiles**	**N (%)**	**Median**	**5-95% Percentiles**	**N (%)**
No bait	females	5	0-10	30 (29%)	7	1-18	49 (77%)
	males	7	0-44	72 (71%)	1	0-8	15 (23%)
CO_2_	females	10	0-20	64 (47%)	8	1-28	59 (84%)
	males	13	5-18	71 (53%)	1	0-6	11 (16%)
MB5 lure	females	7	5-11	44 (40%)	12	2-37	99 (79%)
	males	12	1-20	67 (60%)	4	0-9	26 (21%)
MB5 lure + CO_2_	females	15	11-28	97 (44%)	19	7-23	96 (74%)
	males	16	6-48	121 (56%)	6	0-14	34 (26%)
BG lure	females	14	10-24	93 (44%)	10	0-40	87 (80%)
	males	21	4-34	118 (56%)	2	0-10	22 (20%)
BG lure + CO_2_	females	18	10-32	118 (35%)	18	2-36	113 (74%)
	males	37	5-95	219 (65%)	7	0-19	40 (26%)
Total females				446 (40%)			503 (77%)
Total males				668 (60%)			148 (23%)

Median and 5-95% percentile intervals of *Ae. albopictus* females and males collected with BG-Sentinel traps baited with different odour blends (BG lure and MB5 lure with/without CO_2_) in two areas in Rome (Italy). No bait = negative control.

**Figure 1:**
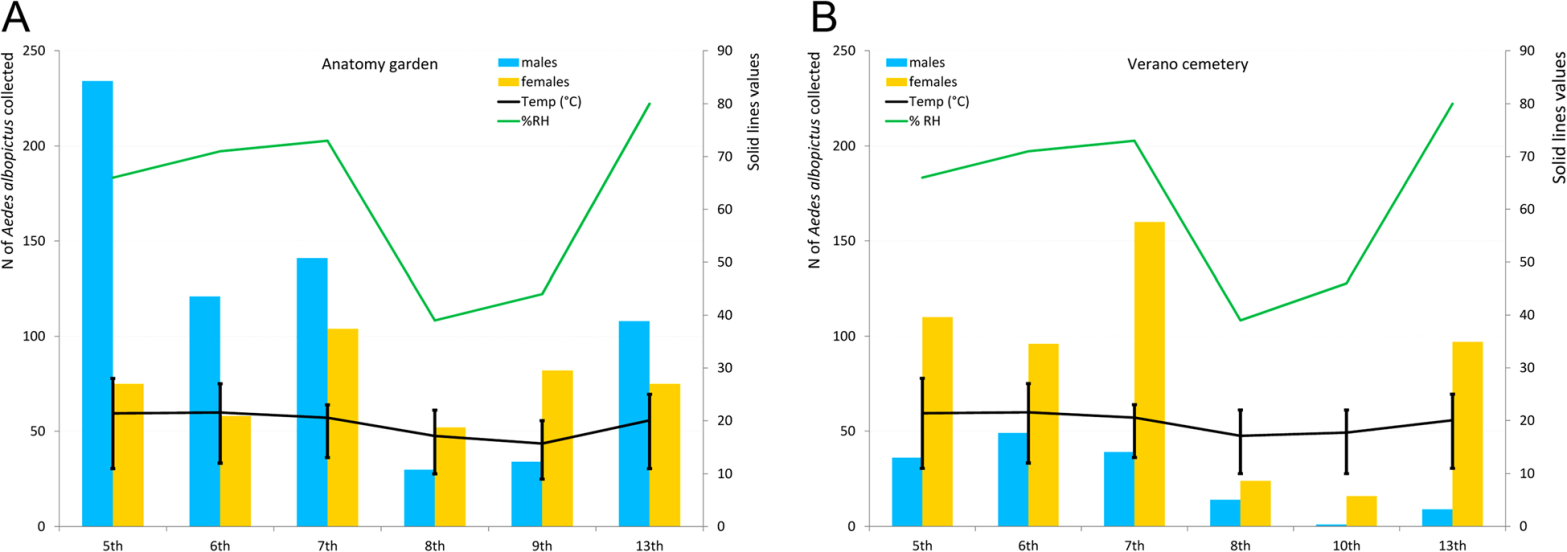
***Aedes albopictus*** **daily collections in relation to relative humidity and temperature.** Total numbers of *Aedes albopictus* males (blue bars) and females (orange bars) daily collected with BG-Sentinel traps in Anatomy garden **(A)** and Verano cemetery **(B)** in relation to mean daily temperature (black solid line, with minimum and maximum) and relative humidity (green solid line). X-axis = dates in October 2011.

While the ambient temperature remained roughly stable during the experiments, fluctuations in daily female collections were positively correlated with relative humidity variations (Pearson’s R^2^ = 0.41, p = 0.026; Figure 1). This phenomenon, already observed in a previous study [21], could be due to a stimulation of host-seeking behaviour by increasing relative humidity, which may be perceived by female mosquitoes as an indication of possible imminent rains and, thus, of increasing availability of oviposition/breeding sites [22]. This hypothesis is also supported by the lack of correlation between daily male collections and relative humidity.

**Figure 2:**
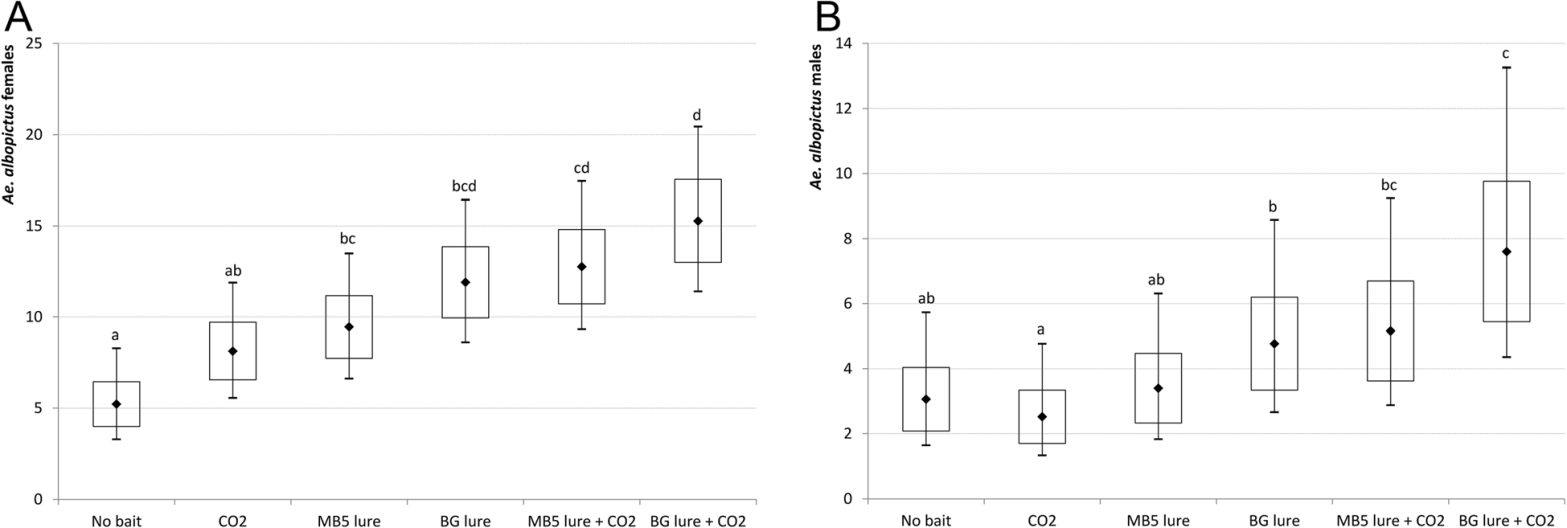
**Daily mean estimates of** ***Aedes albopictus*** **collected with different odour blends.** Daily mean estimates of *Aedes albopictus* females **(A)** and males **(B)** collected with two BG-Sentinel trap-odour blends (BG lure and MB5 lure with/without CO_2_). Solid dots represent the mean estimate for each odour blend. Bottom and top of the box show the standard error range. Whiskers show 5-95% percentile range. Means not sharing the same letter differ significantly at P <0.05. No bait = negative control.

The GLM model for *Ae. albopictus* females retained sampling area (*χ*^2^ = 22.0; p = 0.001), date of collection (*χ*^2^ = 38.3; p < 0.0001) and odour bait tested (*χ*^2^ = 4.39; p < 0.036) as significant effects. BG-trap position was shown not to affect the model significantly, although exposure to sun has been shown to reduce collection efficiency [21]. Significant differences were observed in the mean number of females collected daily in the two sites with traps baited with the different blends (Figure 2A; Table 2): i) all baited traps were significantly more effective than the unbaited ones (t-probabilities of pairwise differences; P < 0.05); ii)traps baited with either lures in combination with CO_2_ were more effective than those baited with CO_2_ alone (MB5 lure + CO_2_ vs CO_2_: P = 0.052; BG lure + CO_2_ vs CO_2_: P = 0.005). No significant differences were observed between traps baited with MB5 lure alone *versus* MB5 lure + CO_2_ and with BG lure alone *versus* BG lure + CO_2_. Finally, no significant differences were observed between the two lures neither in the absence, nor in the presence of CO_2_, although the highest collections were obtained with combinations of each lure with CO_2._

**Table 2:** **Pairwise comparisons of predicted mean estimates of** ***Aedes albopictus*** **collected with different odour blends**

**Females**					
	No bait	CO_2_	MB5 lure	BG lure	MB5 lure + CO_2_
CO_2_	ns				
MB5 lure	0.034	ns			
BG lure	0.02	ns	ns		
MB5 lure + CO_2_	0.001	0.052	ns	ns	
BG lure + CO_2_	0.000	0.005	0.025	ns	ns
**Males**					
	No bait	CO_2_	MB5 lure	BG lure	MB5 lure + CO_2_
CO_2_	ns				
MB5 lure	ns	ns			
BG lure	ns	0.035	ns		
MB5 lure + CO_2_	ns	0.019	ns	ns	
BG lure + CO_2_	0.005	0.002	0.007	0.038	ns

P-values of pairwise comparisons of predicted mean estimates of *Ae. albopictus* females and males collected with different odour blends (BG lure and MB5 lure with/without CO_2_) as obtained by GLM. Only 95% significant p-values are shown. ns = non-significant p-value. No bait = negative control.

GLM model of male collections – of which significant variables are: date of collection (*χ*^2^ = 68.5; p < 0.0001), bait tested (*χ*^2^ = 35.7; p < 0.0001) and trap position (*χ*^2^ = 27.7; p-0.002) - showed a trend only partially consistent with the one observed for females (Figure 2B, Table 2): i) baited traps were not significantly more effective than the not baited ones (probably due to low average catch sizes and large variations in estimates) except for BG lure + CO_2_ (t-probabilities of pairwise differences; P = 0.005), ii) traps baited with BG lure, with or without CO_2_, were more effective than those baited with CO_2_ alone (BG lure vs CO_2_: P = 0.035; BG lure + CO_2_ vs CO_2_: 0.002), whereas MB5 lure was more effective than CO_2_ alone only in combination with CO_2_ (P = 0.019) iii) BG lure showed increased performance when CO_2_ was added to the blend (BG lure + CO_2_ vs BG lure: P = 0.038), whereas no significant differences were observed between traps baited with MB5 lure alone *versus* MB5 lure + CO_2_. The attractive effect of odour blends is likely due to the fact that *Ae. albopictus* males, although not blood-feeding, often mate with females in proximity of potential hosts and thus seek for hosts to increase their reproductive success [18].

In conclusion, the study demonstrates that the MB5 lure – which was developed to specifically attract highly anthropophilic Afrotropical malaria vectors and was shown to be very efficacious in this respect [15] – is as attractive as the BG lure to *Ae. albopictus* despite the generalist feeding habits of this species [12]. This could imply that the MB5 lure can be effectively applied in sampling schemes were both anthropophilic and generalist species are targeted, although its attractiveness to tropical mosquito species remains to be compared to that of the BG lure. The availability of synthetic mosquito lures that are as attractive as a human is highly advantageous as odour-baited traps can be placed across a wide area without the need for operational visits for surveillance purposes. Moreover, recent studies have demonstrated that the residual effect of the MB5 lure exceeds 40 days opening the possibility to leave traps running unattended for several weeks, with a continuous release of attractants [10].

The results show that the addition of the synthetic lures to CO_2_ increases trap performance, thus confirming the importance of this blend combination in mosquito attraction already shown from previous studies with live animals [23]. Addition of CO_2_ to BG lure and MB5 was shown to be highly attractive also to *Ae. aegypti* in the Brazilian Amazon region [24] and in Iquitos, Peru (W. Takken et al., unpublished data), respectively, further supporting addition of CO_2_ as an essential element of synthetic odour baits for mosquitoes. Overall, odour-baited traps, as used in the current study, can be used in epidemiological studies of vector-borne disease risk estimates as well as for establishing the level of mosquito nuisance.

Moreover, the method used to release of CO_2_ by yeast fermentation of sugar was shown to be effective also for *Ae. albopictus* in temperate climates. This method, already representing a cheap and reliable method for collection of other tropical mosquito species, has the clear operational advantage to obviate the more expensive and demanding use of gas tanks or dry ice [13,25].

Finally, the high numbers of males collected in this study show that BG-Sentinel traps baited with either odours in combination with CO_2_ represent an effective tool for measurements of male dispersal, mating behaviour and longevity, which are presently constrained by the lack of efficient sampling tools.

## Conclusions

The results confirm the efficacy of BG-Sentinel trap baited with the standard BG lure and synergized with CO_2_ in collecting not only *Ae. albopictus* females, but also males, whose sampling is increasingly important in the frame of studies aimed to evaluate the potential of sterile insect technique control approaches [26]. Moreover, the results indicate that the MB5 lure, which was developed to attract anthropophilic Afrotropical malaria vectors, is also effective to attract mosquito species, such *Ae. albopictus*, with generalist trophic habits and thus involved in the transmission of human, as well as zoonotic pathogens.

## Abbreviations

MB5: Mbita blend
BG-trap: Biogents BG-Sentinel trap
